# New Insights into Mechanical, Metabolic and Muscle Oxygenation Signals During and After High-Intensity Tethered Running

**DOI:** 10.1038/s41598-020-63297-w

**Published:** 2020-04-14

**Authors:** F. B. Manchado-Gobatto, A. B. Marostegan, F. M. Rasteiro, C. Cirino, J. P. Cruz, M. A. Moreno, C. A Gobatto

**Affiliations:** 1 Laboratory of Applied Sport Physiology, School of Applied Sciences, University of Campinas, Limeira, Sao Paulo Brazil; 2 Postgraduate Program in Human Movement Sciences, Methodist University of Piracicaba, Piracicaba, Sao Paulo Brazil

**Keywords:** Optical spectroscopy, Data acquisition, Metabolism, Respiration, Biomedical engineering

## Abstract

High-intensity exercises including tethered efforts are commonly used in training programs for athletes, active and even sedentary individuals. Despite this, the knowledge about the external and internal load during and after this effort is scarce. Our study aimed to characterize the kinetics of mechanical and physiological responses in all-out 30 seconds (AO30) tethered running and up to 18 minutes of passive recovery. Additionally, in an innovative way, we investigated the muscle oxygenation in more or less active muscles (vastus lateralis and biceps brachii, respectively) during and after high-intensity tethered running by near-infrared spectroscopy – NIRS. Twelve physically active young men were submitted to AO30 on a non-motorized treadmill to determine the running force, velocity and power. We used wearable technologies to monitor the muscle oxygenation and heart rate responses during rest, exercise and passive recovery. Blood lactate concentration and arterial oxygen saturation were also measured. In a synchronized analysis by high capture frequency of mechanical and physiological signals, we advance the understanding of AO30 tethered running. Muscle oxygenation responses showed rapid adjustments (both, during and after AO30) in a tissue-dependence manner, with very low tissue saturation index observed in biceps brachii during exercise when compared to vastus lateralis. Significant correlations between peak and mean blood lactate with biceps brachii oxygenation indicate an important participation of less active muscle during and after high-intensity AO30 tethered running.

## Introduction

Physical exercise performed at different intensities promotes distinct physiological responses during both activity and recovery process^1,2^. High-intensity and short-volume efforts are widely used in the sports context and have also been extensively adopted in non-athlete training programs, such as high-intensity interval training (HIIT) and sprint interval training^3,4^. In the same way, high-intensity tethered exercises (including resisted sled sprinting) performed maximally have been applied to improve physical and athletic performances^5,6^. Despite that, there is a lack of knowledge about the mechanical and physiological kinetics during all-out tethered exercise and recovery. This gap can compromise the training load interpretation when this type of effort is adopted.

Training load is described as external and internal, depending on which measurements of the athlete/participant are assessed^7^. External load is defined as the amount and quality of work performed (e.g. distance covered, velocity and exercise power). On the other hand, the internal load indicates the physiological and psychophysiological responses of the organism to the effort imposed from the external load. However, internal-load indicators, especially during exercise, and the integration of external and internal loads need to be improved^7^.

Most exercise and recovery studies investigate systemic responses to the observed internal load, such as heart rate and blood lactate, the latter being considered a reliable metabolite to indicate the exercise intensity^1^. Although very significant for training direction, so far this metabolite is still often obtained invasively. Furthermore, the blood lactate measurement commonly occurs only a few times during an effort protocol or rest (e.g. only a few points on the timeline), not allowing full monitoring. Lactate is a product of one metabolic pathway (glycolysis) and a substrate for mitochondrial respiration, being regarded as the link between glycolytic and aerobic pathways^8^. This ‘chief messenger’^8^ is more highly produced in more active muscles during high-intensity exercise and is released into the bloodstream; it can be removed during and after physical exercise with important participation of less active muscles in this task. The lactate efflux and influx in skeletal fiber are mediated by MCT4 and MCT1, respectively^9,10^ and there is a dependence on the supply and utilization of oxygen by higher and lower activity muscles during and after exercise, since the lactate removal process is provided by the oxidative pathway in this tissue. Thus, it is possible that the key to maximum physiological equilibrium (i.e., at maximal lactate steady state^1,11^) and even the best chronic adaptations promoted by high-intensity training, is precisely in the muscle responses of more or less active muscles. So, the peripheral respiratory dynamics in exercise and recovery integrated with the mechanical power responses need to be improved, especially in running exercise, due to the extensive use in sports and training programs.

Most investigations involving power efforts and muscle oxygenation analysis during high-intensity exercises were performed on a cycle ergometer^12–15^ and in repeated sprint^13,16,17^ but with measurements conducted in one muscle group^16,18^ or in independent exercise to compare arm vs leg oxygen responses^13^. Rissanen *et al*.^19^ performed simultaneous analysis in biceps brachii and vastus lateralis in incremental treadmill running, observing differences between less and more active muscle oxygenation, especially in severe-intensity exercise. To our knowledge, the monitoring of the external load by mechanical power in a high-intensity running effort or all-out running concurrently with oxygen saturation analysis in two muscles (more and less active) has not yet been investigated. We believe that, in part, this lack is due to the reduced number of protocols/ergometers capable of identifying the precise running power. Additionally, in contrast to more stationary exercise, such as that conducted on a cycle ergometer, in order to analyse the peripheral oxygenation in a running effort (with freedom of limb movement) the use of wearable equipment is desirable.

In the first case our research group developed an innovative ergometer capable of acquiring accurate values of mechanical parameters during running efforts^20–22^ based on the tethered running concept^23,24^. The non-motorized treadmill (NMT) is composed of velocity and force sensors to determine the individual performance in running exercise by the high capture frequency of these signals (1000 Hz)^20,21^. Similar to the classical Wingate test^25^, the all-out 30 seconds (AO30) have been used to identify the force, velocity and power (peak, mean, minimum and fatigue index) in a tethered system^22,26^. However, as it is widely used in training and evaluation programs, the characterization of external and internal load responses during AO30 tethered efforts and in post-exercise still needs to be improved.

The near-infrared spectroscopy (NIRS) technique, purposed at first in 1977^27,28^ is based on the light absorption of oxygenated and deoxygenated hemoglobin and myoglobin in the near infrared tissue, using the interaction of light at different wavelengths^29^. NIRS is a non-invasive method that has been shown to be a significant tool capable of estimating the muscle oxygenation events, such as variations in oxyhemoglobin (O_2_Hb), deoxyhemoglobin (HHb), total hemoglobin (tHb) and tissue saturation index (TSI) in skeletal muscle^30,31^. This technique based on optical principles has been commonly used in clinical studies involving pathologies and exercise prescription^32,33^ and recently focused on inactive participants^12^, active subjects^34,35^ and athletes^16,36–38^ to improve the knowledge about physiological and performance responses. In a recent systematic review, Perrey and Ferrari^39^ suggested that the popularity of muscle oxygenation studies in exercise increased after the commercialization of portable wireless muscle oximeters. In this context, by allowing continuous and sensitive monitoring with high frequency of physiological signal capture, the use of NIRS − potentially and in the near future − may contribute to the improvement of the organization of exercise and training load monitoring aimed at improving health and performance.

Considering the significant application of high-intensity tethered exercises in training programs and the knowledge gap regarding the acute responses during and after this effort, our study aimed to characterize the kinetics of mechanical and physiological responses in all-out 30 s running effort and up to 18 minutes of passive recovery after this kind of exercise. Additionally, in an innovative way, we investigated the muscle oxygenation in more or less active muscles (vastus lateralis and biceps brachii, respectively) during and after high-intensity tethered running using wearable NIRS. Based on a previous study using an incremental running test^19^, we hypothesize, there will be a significant difference between arm and leg oxygenation during and after an all-out 30 second tethered running effort. Additionally there will be a significant relationship between muscle oxygenation and blood lactate responses.

## Methods

### Subjects

Twelve physically active young men were evaluated (22 ± 1 years, body mass 71.4 ± 2.7kg, height 178 ± 2 cm). Subjects answered the International Physical Activity Questionnaire, in which the minimum score to classify them as ‘physically active’ was used as inclusion criterion^40^. All subjects reported no metabolic, cardiovascular or orthopaedic disease and no use of medications or drugs. This study was conducted in agreement with the ethical recommendations of the Declaration of Helsinki and all experiments were approved by the Research Ethics Committee of The School of Medical Sciences (protocol number 99783318.4.0000.5404). After having received information about the experimental procedures and risks, all individual participants signed an informed consent form.

### Experimental design

**Figure 1:**
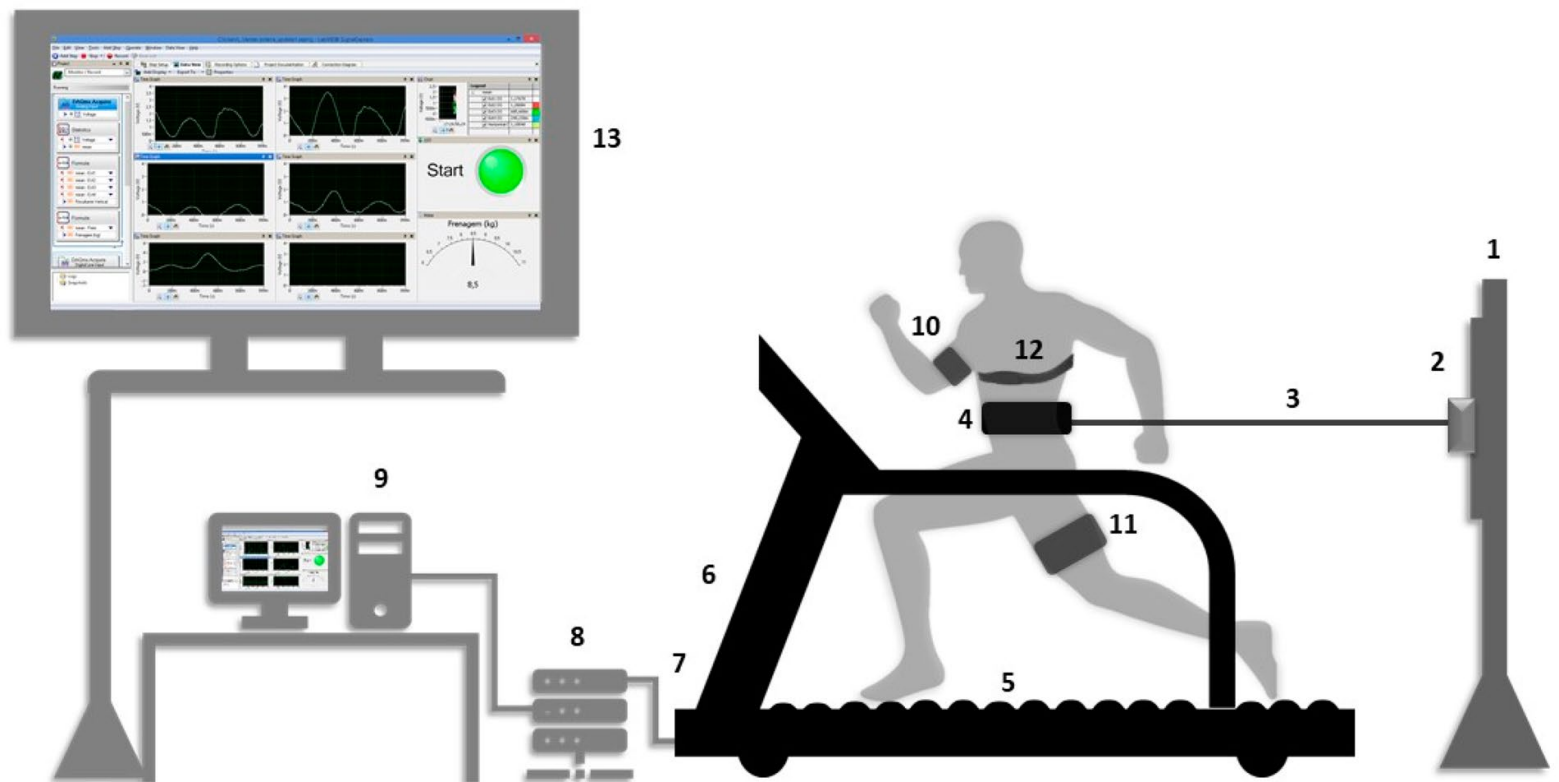
(**1**) Height adjustment bar; (**2)** load cell; (**3)** steel cable; (**4)** belt attached to the steel cable and participant; **(5)** sensorized platform by four load cells; (**6)** non-motorized treadmill (NMT); **(7)** Hall-effect sensor positioned on the treadmill’s front cylinder to determine the velocity that results from the connection with the acquisition system; **(8)** acquisition system composed of portable amplifier (MKTC5-10, MK) and an acquisition module (NI USB-6009); **(9)** computer; **(10)** NIRS *Portamon* unit fixed to the upper limb (biceps brachii muscle); **(11)** NIRS *Portamon* unit fixed to the lower limb (vastus lateralis muscle); **(12)** heart rate monitor (Polar V-800), **(13)** monitor showing the force, velocity and power during tethered test (online signals).

The experimental design consisted of three laboratory visits, separated by 24–48 h. Firstly, subjects received information about the experimental design and signed a consent form. Subjects answered the International Physical Activity Questionnaire (IPAQ) and a questionnaire for health characterization. Next, participants were submitted to anthropometric and body composition measurements^41^. On the second day, the tethered running familiarization was conducted on a non-motorized treadmill (NMT). Subsequently, during the third day, subjects were submitted to AO30s tethered running test to determine running force, velocity and power. The participants were also equipped with wearable technology (NIRS on the upper limb and lower limb and a heart rate monitor) to obtain muscle oxygenation and heart rate responses, respectively. Ergometer specifications and equipment are shown in Fig. 1. After the AO30 running test, participants remained at rest for 18 minutes to capture of physiological and muscle responses. Rest in the dorsal decubitus position was adopted to minimize the discomfort experienced by active individuals after all-out efforts.

### Exercise test protocol (AO30) and post-exercise (recovery)

All procedures were conducted in a controlled laboratory (temperature = 22 °C ± 1 °C (SD); relative humidity = 50% ± 2%; luminosity = ~300 lx). To perform the AO30 test conducted in the third session, participants were equipped with wearable equipment (NIRS and heart rate monitor) and kept in rest (sitting position) during a 3 minute period aimed to establish baseline responses. After this, before the AO30 test, the subjects warmed up on a motorized treadmill running (Inbramed Super ATL, Inbrasport, Brazil) for 5 minutes at 7.0 km/h. After the warm-up, 5 minutes of recovery was taken to return the physiological responses to rest. Then the AO30 tethered running was carried out with mechanical and physiological responses recorded (muscle oxygenation and HR). The participants received constant verbal encouragement during the tethered test exercise.

**Figure 2:**
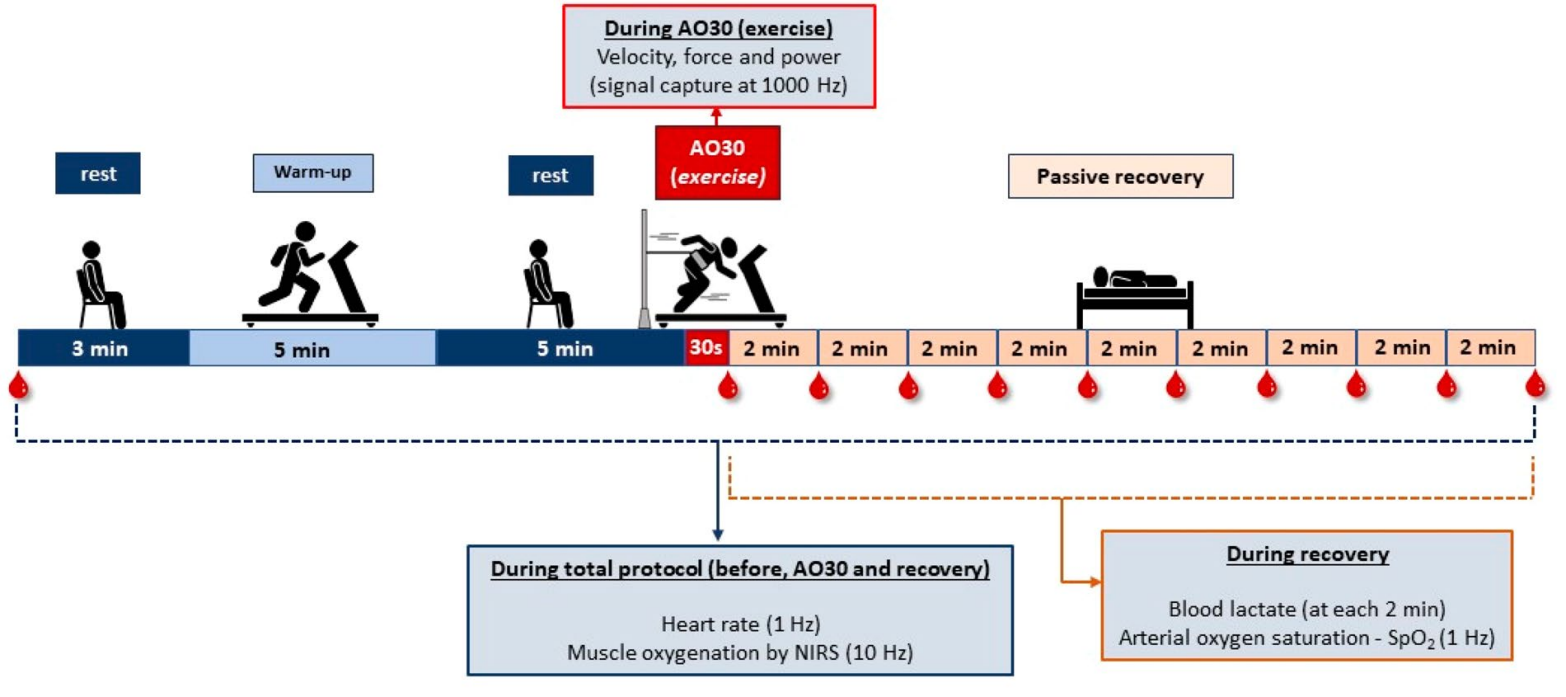
Experimental high-intensity exercise session adopted to test the mechanical and physiological responses, and muscle oxygenation in upper and lower limb.

Immediately after the exercise, a pulse oximeter was plugged on to the subject’s finger and blood capillary samples were extracted from the ear lobe by heparinized capillary tubes. With the aim of conducting the recovery investigations, HR, arterial oxygen saturation (SpO_2_) and muscle oxygenation were continuously monitored for up to 18 minutes after AO30, with blood capillary samples extracted (ear lobe) immediately and every two minutes during this recovery period. The timeline of the AO30 test procedures and recovery are explained in Fig. 2.

### Mechanical measurements in AO30

The AO30 tests were performed on a standardized non-motorized treadmill^20–22^. Subjects ran with an inextensible steel cable in series with a load cell (CSL/ZL-500, MK Controle e Instrumentação Ltda, Brazil) attached to their waist for a directly horizontal force measurement^22^. Vertical force during the running test was also captured by four load cells positioned under a platform (NMT). Velocity was obtained as the first derivative of the treadmill displacement using a Hall-effect sensor. Thus, power was obtained by the product between force and velocity. The acquisition system consisted of a strain gauge (CSA/ZL-500 MK Control, Sao Paulo, Brazil), a portable amplifier (MKTC5–10, MK Control, Sao Paulo, Brazil) and an acquisition module (NI USB-6009, National Instruments, Austin, USA). Mechanical measures were captured via signals (LabView Signal Express 2009 National Instruments®) with 1000 Hz acquisition. The NMT system was calibrated daily^21^ modulated and subsequently transferred to MatLab (R2008a MatLab®, MathWorkstm). Peak, mean and minimum of the force and power were displayed in absolute and relative body mass values.

### Muscle oxygenation measurements and analyses

Changes in the muscle oxygenation were assessed continually during the rest, exercise and recovery periods using a NIRS. For this, two PortaMon devices (Artinis Medical Systems BV, Zetten, Netherlands) including three light source transmitters (each one with two wavelengths between 750 and 850 nm) at 30, 35, and 40 mm distance to the receiver, were used to determine the tissue saturation index (TSI, %). These devices were also used to obtained the changes in the oxyhemoglobin ([O_2_Hb]), deoxyhemoglobin ([HHb]) and total hemoglobin ([tHb]) concentrations by analysis of the deeper trace (deepest optode, 40 mm).

These NIRS devices were positioned on two right muscles: the biceps brachii (BB), in medial biceps brachii portion^14,42,43^, an upper limb muscle considered as less active during running exercise, and the vastus lateralis belly (VL), 15 cm above the proximal edge of the patella and 5 cm towards the external side^44,45^ parallel to the long axis of the muscle^19^ considered more active during running effort^16,46^.The equipment was wrapped tightly in a transparent plastic to avoid humidity and which created a waterproof barrier^13^, and by a dark band, to secure the probe and protect it from environmental light.

The concentration values of muscle oxygen were analysed by entering the differential pathlength factor (DPF) values. According to the manufacturer’s instructions, the DPF for different tissues and participant characteristics must be selected according to the literature. In this sense, considering the PortaMon NIRS equipment, several studies analysing the vastus lateralis muscle applied DPF (range of 3.7 to 4.16 DPF) for participants and experimental designs with similar characteristics to our study^39^. We, therefore, adopted 3.83 to VL^47,48^. On the other hand, there is a lack of information about DPF for biceps brachii^13^. In the present study, we applied DPF 3.78 to BB, based on Fauss *et al*.^49^, which used similar values to triceps brachii, and also on Duncan^50^, which measured DPF in male adult arms.

The signal was captured at 10 Hz. After signal capture, the data were smoothed using the 10^th^ order low- pass-zero phase Butterworth filter (cut-off frequency 0.1 Hz)^16^ provided by recording and analysis Artinis software (Oxysoft, Artinis Medical System, Netherlands). The change (Δ) in [O_2_Hb], [HHb], and [tHb], in micromolar units (µM), were obtained by subtracting these values from the baseline data^12,51^, considering the final 30 seconds of the baseline period^17^ of 3 minutes (Fig. 2).

### Heart rate, arterial oxygen saturation and blood lactate concentration

Heart rate was continuously recorded every 1 second by a heart rate monitor (Polar V800, Finland). Blood samples (25 µl) were collected at rest and during post-exercise from the ear lobe with a heparinized capillary, and deposited into microtubes (Eppendorf, 1.5 ml) containing 50 µl of 1% sodium fluoride (NaF). The samples were frozen at –20 °C before being homogenized and determined by a lactate analyser (YSI-2300-STAT-Plus™, Yellow Springs, USA). Estimations of arterial oxygen saturation were accomplished immediately after AO30 and during all recovery phase with a pulse oximeter (OXIFAST Takaoka, SP, Brazil).

### Statistical analysis

All results are expressed as mean ± error of the mean (SEM). Distribution of the normality and variance homogeneity were initially tested by the Shapiro−Wilk and Levene test, respectively. One-way repeated measures analysis of variance (ANOVA) was applied to study the effect of the time during the exercise (on mechanical and physiological responses) and during of the recovery phase (on physiological responses). A two-way analysis of variance (ANOVA) for repeated measures (effects of time and site device NIRS) investigated the difference between upper limb (BB) and lower limb (VL) muscle oxygenation (Δ[O_2_Hb], Δ[HHb], Δ[tHb], and TSI) in AO30 (at each 1 s) and post-exercise (immediately after exercise and at each 2 minutes until 18 minute). Newman−Keuls post hoc test was used to detect these differences. Paired Student’s t-test was applied to assess the difference between peak, mean and minimum responses of local tissue oxygenation. The relationship among mechanical, physiological and muscle oxygen responses in exercise and recovery were obtained by Pearson’s linear regression test. All statistical analyses were performed using STATISTICA software (7.0 version). Considering that this is the first methodological study to characterise the mechanical, physiological, and muscle oxygenation in AO30 in tethered running and in the line of exercise and physical training studies, statistical significance was set at P ≤ 0.05.

## Results

The participants characterization is expressed in Table 1.

**Table 1:** Participants’ characteristics (n = 12).

Participants’ characteristics	
	Mean ± SEM
Age (year)	22 ± 1
Body mass (kg)	71.4 ± 2.7
Height (cm)	178 ± 2
Body fat percentage (%)	8.7 ± 1
Vastus laterais skinfold (mm)	13.0 ± 1.4
Biceps brachii skinfold (mm)	3.7 ± 0.2
HR rest (bpm)	74 ± 3
[Lac] rest (mM)	0.9 ± 0.1
SpO_2_ rest (%)	98.1 ± 0.2

HR-heart rate; [Lac] - blood lactate concentration; SpO_2_- arterial oxygen saturation.

### Exercise responses

**Figure 3:**
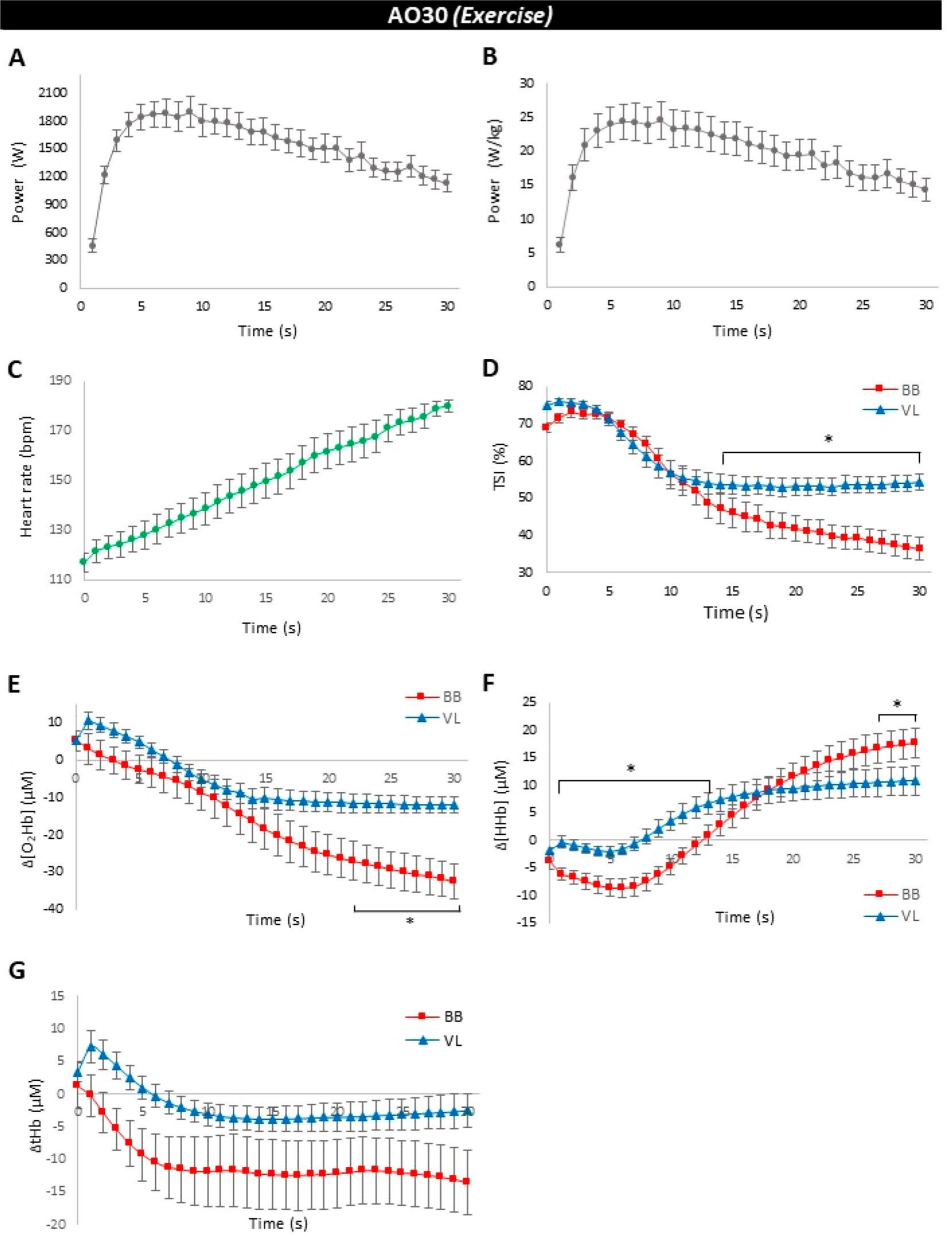
Results obtained during all-out 30 s (AO30 exercise) at tethered running in a non-motorized treadmill (mean ± SEM at each 1 s, n = 12). (**A**) Absolute running power (W), (**B**) Relative running power (W/kg), (**C**) Heart rate (bpm), (**D**) Tissue saturation index (TSI) (%) in biceps brachii (BB) and vastus lateralis (VL), (**E**) Changes (ΔµM) in oxyhemoglobin ([O_2_Hb]) occurred in BB and VL, (**F**) Changes (ΔµM) in deoxyhemoglobin ([HHb]) in BB and VL, and (**G**) Changes (ΔµM) in total hemoglobin ([tHb]) occurred in BB and VL, measured by near infrared-spectroscopy (NIRS). *Indicates the difference between responses obtained in BB and VL at the same moment (P ≤ 0.05).

The main values (peak, mean and minimum results) of the mechanical parameter obtained by AO30 tethered running are summarized in Table 2. The curves of absolute and relative power output during AO30 test are shown in Fig. 3 (Panels A and B). We observed strong and significant relationships between absolute peak power and relative peak power (r = 0.87, P = 0.000). Peak power also showed a significant correlation between mean power (r = 0.81, P = 0.001) and relative mean power (r = 0.97, P = 0.000), absolute peak and mean force (r = 0.95, P = 0.000 and r = 0.92, P = 0.000, respectively) and peak and mean velocity (r = 0.83, P = 0.001 and r = 0.71, P = 0.001, respectively). Repeated measures ANOVA one-way showed an effect of time of test on absolute power (F = 5.95, P = 0.000) and relative power (F = 9.37, P = 0.000). Both, relative and absolute power increased during the first seconds of the test, and attained peak values around the 6^th^ second of tethered running (Fig. 3, Panel A and B). After that time, there was a reduction in the power values, with significant difference among the last seconds of the test (29^th^ and 30^th^ s) compared to 6–9 seconds (P ≤ 0.05). Fatigue index (FI = (peak power − minimum power)/peak power *100) was 47.0 ± 2.7% (Table 2).

**Table 2:** Peak, mean and minimum absolute and relative (to body mass) mechanical results obtained during tethered running exercise (AO30) on a non-motorized treadmill (NMT) (n = 12).

Mechanical Parameters	
*AO30s – Exercise*	
	Mean ± SEM
**Power**	
Peak power (W)	2017.8 ± 159.7
Mean power (W)	1514.9 ± 119.6
Minimum power (W)	460.8 ± 59.5
Peak power (W/kg)	28.2 ± 1.6
Mean power (W/kg)	21.2 ± 1.3
Minimum power (W/kg)	6.6 ± 0.9
**Force**	
Peak Force (N)	427.9 ± 23.1
Mean Force (N)	365.9 ± 20.9
Minimum Force (N)	251.8 ± 19.1
Peak Force (N/kg)	6.0 ± 0.3
Mean Force (N/kg)	5.2 ± 0.3
Minimum Force (N/kg)	3.6 ± 0.3
**Velocity**	
Peak Velocity (m/s)	5.0 ± 0.1
Mean Veocity (m/s)	4.1 ± 0.1
Fatigue index (%)	47.0 ± 2.7

Figure 3 shows the curves of heart rate (Panel C), TSI (Panel D), deltas of oxyhemoglobin (Δ[O_2_Hb]), deoxyhemoglobin (Δ[HHb]) and total hemoglobin (Δ[tHb]) (Panels E, F and G, respectively) during 30 s of tethered running. HR was affected by time (F = 9.26, P < 0.001), increasing throughout the AO30 test, with higher and significant values observed after 16^th^ second compared to the first seconds of exercise. The last HR value (HR in 30^th^ second) was higher than HR at the 1^st^ to 15^th^ s of test and the peak, mean and minimum HR values were 180 ± 2, 151 ± 6 and 121 ± 5 bpm, respectively.

A two-way repeated measures ANOVA presented the effect of time of test (F = 29.21, P < 0.001), site device NIRS (F = 109.78, P < 0.001) and interaction between time x site device NIRS (F = 2.99, P < 0.001) to TSI. In the upper limb (BB) the percentage of tissue saturation decreased during the initial 10 seconds of AO30 (significant difference compared to the 1^st^ second exercise from the 9^th^ test seconds), and then showed stabalisation in lower values. The same results were observed by TSI in VL, but a significant decrease occurred from the 7^th^ second of the exercise. When comparing the TSI response in BB and VL at the same times of the test, a greater drop was observed in BB, with different results after the 14^th^ second. Higher TSI values in more active muscle in our experimental conditions (VL) were observed during exercise.

With regard to oxygen availability (Δ[O_2_Hb]) and oxygen utilization (Δ[HHb]), an inverse curve behaviour was observed, as expected (Panels E and F). There is an effect of time (F = 12.17, P < 0.001 and F = 24.27, P < 0.001) and site of NIRS (F = 121.31, P < 0.001 and F = 7.96, P = 0.005) for Δ[O_2_Hb] and Δ[HHb], with significant interaction between time x site NIRS to Δ[HHb] (F = 3.61, P < 0.001), but not to Δ[O_2_Hb] (F = 1.08, P = 0.351). Delta BB Δ[O_2_Hb] response presented a drop during all exercise, but decreased significantly after 17 second of the test. In the leg muscle (VL), this delta was significantly reduced after 7^th^ second, with stabilization of Δ[O_2_Hb] after the 14^th^ second. The comparison between Δ[O_2_Hb] responses in BB and VL at the same test times revealed only significant differences in the last 8 seconds of the AO30 (22–30 seconds), with more oxygen available to leg muscle (VL) (P ≤ 0.05) (Fig. 3, panel E). Delta of [HHb] apparently reduced during the first exercise seconds for both, BB and VL muscles. In the arm (BB), higher values of delta [HHb] were observed after 14 second of the exercise, and a drop after 18 second compared to 1–7 seconds for the leg (VL). There was no significant difference of Δ[tHb] between BB and VL during the AO30 exercise, but greater interindividual variation was visualized in the upper limb (P < 0.05).

### Recovery responses

**Figure 4:**
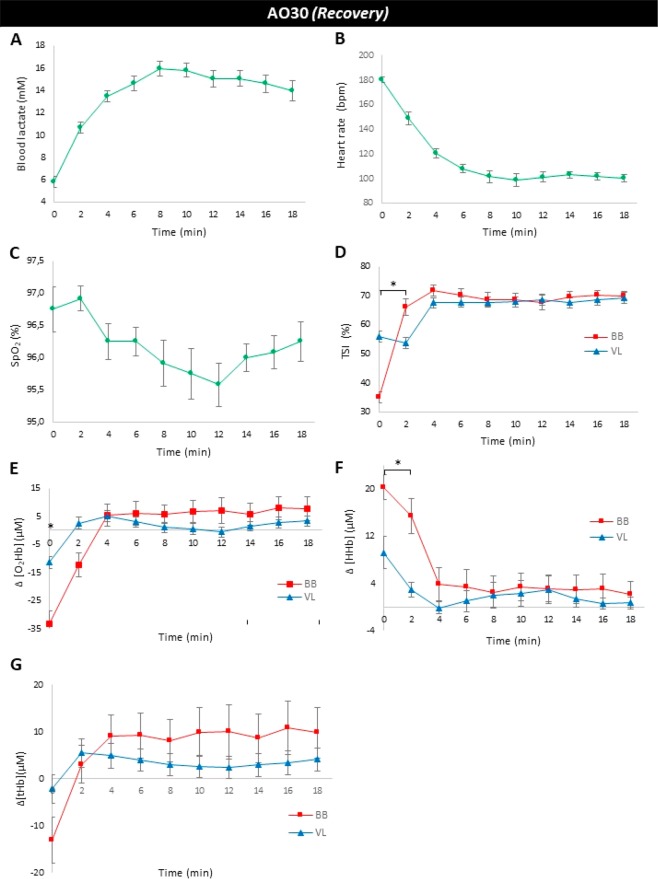
Results obtained after all-out 30 s (AO30 recovery) at tethered running in a non-motorized treadmill (mean ± SEM at end and each 2 min during passive recovery, n = 12) **(A)** Blood lactate concentration (mM), **(B)** Heart rate (bpm), **(C)** arterial oxygen saturation – SpO_2_ (%), **(D)** Tissue saturation index (TSI) (%) in biceps brachii (BB) and vastus lateralis (VL), **(E)** Changes (ΔµM) in oxyhemoglobin ([O_2_Hb]) occurred in BB and VL, **(F)** Changes (ΔµM) in deoxyhemoglobin ([HHb]) in BB and VL, and (**G)** Changes (ΔµM) in total hemoglobin ([tHb]) occurred in BB and VL, measured by near infrared-spectroscopy (NIRS). *Indicates the difference between responses obtained in BB and VL at the same moment (P ≤ 0.05).

Table 3 and Fig. 4 summarize the main results of the physiological responses after AO30 in tethered running. Repeated one-way measures ANOVA showed effect of time on recovery of HR (F = 45.2, P = 0.000) and blood lactate (F = 20.49, P = 0.00), but not on SpO_2_ (F = 2.0, P = 0.055). The peak HR in the recovery phase occurred at 180 ± 2 bpm, which is equivalent to 91% of HR_max_ predicted by age (HR_max_ = 220-age). The HR curve showed a decrease in values after 2 minutes post-exercise. Despite lower results visualized during post-exercise, HR at 18^th^ minute did not return to baseline value (100 ± 3 bpm vs 74 ± 3 bpm, respectively) (Fig. 4, Panel B). Immediately after AO30, the blood lactate started to increase (Fig. 4, Panel A) and the peak lactate (peak [Lac]) was attained individually at 8–10 minutes of the passive recovery, at 16.2 ± 0.7 mM. In addition, the rate of blood lactate recovery ((peak[Lac] – minimum [Lac])/peak [Lac]*100) was 64.4 ± 2.3%, which contributed to the partial removal of lactate observed at the end of AO30 (13.9 ± 0.9 mM) but not a return to the baseline concentration (0.94 ± 0.13 mM). SpO_2_ was not significantly modified during our protocol (Fig. 4, Panel C).

**Table 3:** Peak, mean and minimum values of the heart rate (bpm), blood lactate concentration (mM), time to reach the peak of blood lactate concentration (min), rate of blood lactate recovery ((peak [Lac]–minimum [Lac])/peak [Lac] *100) (%) and arterial oxygen saturation (%) during post-exercise (AO30 recovery) (n = 12).

Physiological Responses	
*AO30s – Recovery*	
	Mean ± SEM
**Heart rate**	
Peak HR (bpm)	180 ± 2
Mean HR (bpm)	116 ± 3
Minimum HR (bpm)	95 ± 5
**Blood lactate**	
Peak [Lac] (mM)	16.2 ± 0.7
Mean [Lac] (mM)	13.5 ± 0.6
Minimum [Lac] (mM)	5.8 ± 0.5
Time to peak [Lac] (min)	9.2 ± 0.5
[Lac] rate (%)	64.4 ± 2.3
**SpO**_**2**_	
Peak SpO_2_ (%)	97.1 ± 0,3
Mean SpO_2_ (%)	96.2 ± 0.2
Minimum SpO_2_ (%)	95.2 ± 0.3

HR-heart rate; [Lac] - blood lactate concentration; SpO_2_ – arterial oxygen saturation.

Muscle oxygenation curves in upper and lower limb are expressed in Fig. 4 (panels D–G). Two-way ANOVA for repeated measures presented the effect of time of test (F = 30.10, P < 0.001), site device NIRS (F = 108.99, P < 0.001) and interaction between time × site NIRS (F = 5.23, P < 0.001) to TSI. Differently from that observed with HR and blood lactate concentration, TSI in both muscles increased after the end of exercise, but in BB, this phenomenon was faster than VL. After the 4^th^ minute of recovery, there were no differences between TSI in BB and VL, with values returning to close to those at rest.

In the recovery phase, oxy- and deoxyhemoglobin in both tissues (BB and VL) returned to initial values after 4 minutes of rest (Fig. 4, Panels E and F). When comparing the muscle oxygenation recovery curves to upper and lower limb tissues, we observed significant differences between BB and VL only at the first 4 minutes of recovery (to oxy- and deoxyhemoglobin), with BB showing re-oxygenation more quickly compared to VL. Delta of the total hemoglobin (Δ[tHb]) was not altered in BB and VL during the recovery phase, and the two-way ANOVA for repeated measures showed an effect of time (F = 2.46, P = 0.011) but did not indicate effects of site NIRS (F = 3.60, P = 0.590) and interaction between time × site NIRS (F = 1.11, P = 0.354) for this response.

In addition, the comparison and relationships between peak, mean and minimum values of the TSI and delta of oxyhemoglobin, deoxyhemoglobin and total hemoglobin in the biceps brachii and vastus lateralis (during exercise and recovery) are shown in Table 4. There were significant differences between TSI (mean and minimum values) in BB and VL during AO30, as well as minimum Δ[O_2_Hb] and peak and minimum Δ[HHb] between BB and VL muscles. After AO30 (recovery phase), we observed higher values of TSI in more active muscle (VL) compared to less active muscle (BB). Despite this, total hemoglobin was not significantly influenced by the recovery phase.

**Table 4:** Peak, mean and minimum values of the muscle oxygenation (tissue saturation index and ΔµM of oxyhemoglobin [O_2_Hb], deoxyhemoglobin [HHb] and total hemoglobin [tHb]) in biceps brachii (BB) and vastus lateralis (VL) muscles in AO30 (exercise and recovery). Additionally, this table shows the differences and  correlations between muscle oxygenation in BB and VL (n = 12).

Muscle Oxygenation				
	*BB*	*VL*	*BB vs VL*	
	*Mean* ± *SEM*	*Mean* ± *SEM*	*P value*	*r value (P value)*
***AO30s – Exercise***				
**TSI**				
Peak (%)	76 ± 2	79 ± 2	0.230	0.02 (0.939)
Mean (%)	52 ± 2	60 ± 2	0.010	0.30 (0.331)
Minimum (%)	31 ± 3	50 ± 4	0.000	0.55 (0.062)
**Δ[O**_**2**_**Hb]**				
Peak (µM)	8 ± 3	8 ± 2	0.991	−0,71 (0.825)
Mean(µM)	−16 ± 4	−8 ± 2	0.160	−0.56 (0.058)
Minimum (µM)	−33 ± 5	−13 ± 2	0.007	−0.36 (0.241)
**Δ[HHb]**				
Peak (µM)	19 ± 2	10 ± 3	0.001	0.69 (0.013)
Mean (µM)	5 ± 2	6 ± 2	0.613	0.48 (0.117)
Minimum (µM)	−10 ± 2	−2 ± 1	0.003	−0.16(0.629)
**Δ[tHb]**				
Peak (µM)	3 ± 4	7 ± 3	0.406	−0.47 (0.125)
Mean (µM)	−11 ± 5	−2 ± 2	0.131	−0.28 (0.383)
Minimum (µM)	−18 ± 5	−5 ± 2	0.039	−0.23 (0.478)
***AO30s – Recovery***				
**TSI**				
Peak (%)	69 ± 2	75 ± 1	0.027	−0.46 (0.134)
Mean (%)	61 ± 2	71 ± 2	0.005	−0.50 (0.096)
Minimum (%)	35 ± 2	56 ± 2	0.000	0.37 (0.231)
**Δ[O**_**2**_**Hb]**				
Peak (µM)	10 ± 5	6 ± 2	0.503	−0,39 (0.213)
Mean(µM)	1 ± 4	1 ± 2	0.988	−0.82 (0.001)
Minimum (µM)	−33 ± 5	−11 ± 2	0.003	−0.36 (0.250)
**Δ[HHb]**				
Peak (µM)	22 ± 2	9 ± 3	0.000	0.49 (0.109)
Mean (µM)	6 ± 2	2 ± 2	0.207	0.16 (0.628)
Minimum (µM)	0 ± 2	−1 ± 1	0.927	−0.09 (0.785)
**Δ[tHb]**				
Peak (µM)	14 ± 6	7 ± 3	0.361	−0.27 (0.388)
Mean (µM)	7 ± 5	3 ± 3	0.574	−0.35 (0.261)
Minimum (µM)	−14 ± 4	−3 ± 3	0.052	−0.07 (0.819)

Results are expressed by mean ± standard error of the mean (SEM). Significance was pre-fixed at P ≤ 0.05.

In order to analyse the relationship between mechanical, physiological and muscle oxygenation in more or less active muscle, the Pearson linear regression test was applied. We did not observe a significant relationship among mechanical parameters (force, velocity and power) with physiological and muscle responses. On the other hands, significant correlations were observed between fatigue index with blood lactate and muscle oxygenation on BB. FI was significantly correlated with peak, mean and minimum [Lac] (r = 0.63, P = 0.027; r = 0.66, P = 0.020, and r = 0.68, P = 0.015, respectively), and it was inversely correlated with peak and mean Δ[tHb] in BB during AO30 exercise.

We chose to observe the correlations considering the points of the timeline close to the time of reaching the peak of lactate (i.e., in the 8^th^ and 10^th^ minutes of the recovery). In fact, although the BB and VL muscle oxygenation responses are similar in comparative analysis, the peak [Lac] showed only significant correlation with TSI and Δ[HHb] in BB at the 8^th^ minute (r = 0.62, P = 0.028 and r = −0.75, P = 0.008, respectively) and at the 10^th^ minute (r = 0.63, P = 0.028 and r = −0.77, P = 0.003, respectively), but not with the same responses in the VL. Adopting the minimum, mean and peak values to the muscle responses (as shown in Table 4), we observed an inverse relationship between peak [Lac] with minimum Δ[HHb] in biceps brachii during exercise (r = −0.61, P = 0.034), mean and minimum Δ[HHb] in recovery phase (r = −0.79, P = 0.002; and r = −0.86, P = 0.001, respectively) and mean and minimum TSI values in BB after exercise (r = 0.58, P = 0.046, and r = 0.66, P = 0.019). Still in this way, mean [Lac] showed an inverse and significant correlation with mean and minimum Δ[HHb] in BB at post-effort (r = −0.77, P = 0.03, and r = −0.82, P = 0.030), and with a mean of TSI in BB during recovery phase (r = 0.64, P = 0.025). Relationships between this metabolite and vastus lateralis oxygenation were only observed by peak [Lac] with mean Δ[O_2_Hb] (r = 0.58, P = 0.46) and mean [Lac] with mean and minimum Δ[tHb] values (r = 0.66, P = 0.019, and r = 66, P = 0.020, respectively), both during AO30 responses.

## Discussion

The main highlight of the study was purposing a synchronized form to investigate the mechanical, physiological and oxygenation responses in more or less active muscles during and after high-intensity exercise (AO30) in tethered running conditions. To the best of our knowledge, this is the first study to evaluate muscle oxygen responses in this type of exercise. Here, we used robust tools to obtain power running in an anaerobic effort using high-frequency signal capture and muscle oxygenation measurements with NIRS of the upper and lower limb. The NIRS used here were characterized by wearable and wireless technology currently at the frontiers of knowledge in exercise physiology. We are certain that it is necessary to improve the understanding of the interaction among physiological signals observed during and after high-intensity effort, as in tethered exercises.

The choice to analyse running exercise was due to the importance of this motor skill in high-intensity efforts employed in the sports modality. Our choice to use the tethered system was based on the path to allow quantification of the running power, since tethered running training and sled training have been frequently used in sports evaluation^52,53^, and physical training programs aimed at improving velocity and power^54,55^.

Regarding the mechanical responses throughout the AO30, we measured both vertical and horizontal force components during the running exercise to calculate the power run (Fig. 1). The current findings of peak and mean power (Table 2 and Fig. 3, Panels A and B) were higher than those observed by other authors who used the same ergometer to evaluate recreational endurance runners^26^ and young soccer player athletes in this motorized treadmill^56^, but using only the horizontal force component. In this sense and to check this, when analysed at the same form of the cited authors, the peak and mean powers (peak power = 714.4 ± 35.0 W and 10.0 ± 0.3 W/kg) were similar to those observed by them. The peak power (Fig. 3, panels A and B) was obtained around 6 second at the AO30, and the curve fit behaviour in tethered running was similar to that observed in another 30 s all-out kind of exercise, such as power responses in cycle ergometer^25^ and force curve in swimming^57^ and kayak effort^58^. In contrast, the peak of running velocity in our experiment was obtained within a lower time than that recently observed by Morrison *et al*.^17^ who submitted amateur athletes to four series of AO4s sprints in a non-motorized treadmill (Woodway, Waukesha, Wisconsin). These differences can be attributed to the sample characteristics, time of effort and the use of the tethered run, in our experimental design.

Classically, all-out efforts like 30 s have been used to evaluate mechanical power and the anaerobic system efficiencies^59,60^. The physiological responses during and after AO30 tethered running confirm the high-intensity nature of this exercise to our active subjects, as it occurs, for example, in sprint interval exercise^12,48^. Within a single short duration bout (30 s), HR reached near maximum values (Fig. 3, Panel B) and the exercise showed a lactic anaerobic characteristic, with peak lactate concentration reaching high values (16.3 ± 0.7 mM). These physiological responses did not return to the rest values even after 18 minutes of recovery (Fig. 4, Panels A and B). In contrast, as observed by Morrison *et al*.^17^ in repeated treadmill sprints, the arterial saturation oxygenation (SpO_2_) was not altered by AO30 (Fig. 4, panel C).

In 2011, Ferarri, Muthalib and Quaresma^30^ conducted an interesting review aimed at understanding the skeletal muscle physiology using NIRS technology. Thus, years ago these authors suggested as a possible future direction the association of the muscle oxygenation measurement with other physiological responses monitored during tests and training (for example, HR and blood lactate concentration). Recently, this way has been accomplished and some studies aimed to adopt these recommendations^16,18^.

However, despite the significance of tissue oxygenation^18^ there are very few studies investigating the more and less active muscle responses during and after exercise^14,42,61–63^ especially in running effort^12,19^. According to Perrey and Ferrari in a recent review^39^, the majority of the NIRS studies examined the responses of the vastus lateralis muscle. On the other hand, we believe that knowledge about simultaneous muscle oxygenation in different tissues can provide the potential to improve comprehension of the internal load, especially in dynamics of exercise:rest ratio prescription in physical and sport training programs.

Based on our current knowledge, there is an important lack of investigations evaluating the Δ[HHb] or TSI in simultaneous analyses of arm and leg muscles during and after high-intensity running exercise^19^, and in all-out tethered running. Thus, our main results on muscle oxygenation during AO30 were the differences shown between tissue saturation (TSI) in biceps brachii (BB) and vastus lateralis (VL) (Fig. 3, panel D). Although the TSI decreased significantly for both muscles studied here, there was a greater drop in the biceps brachii (from ~70% to ~35%) compared to the VL responses (from ~75% to ~55%) suggesting an integrative physiological adjustment to maintain the more active musculature during high-intensity exercise, at least in short duration all-out tethered running. In biceps brachii, TSI at the end of all-out effort was very low (35 ± 2%) (Fig. 3 and Table 4), which is what strengthens this hypothesis regarding the difference between leg and arm muscles in AO30 tethered running.

Our results corroborated a previous study involving simultaneous biceps brachii and vastus lateralis during high-intensity incremental treadmill exercise^19^ Rissanen *et al*.^19^ investigated the BB, VL and alveolar gas exchange of healthy male volunteers submitted to an incremental running effort (started at 8 km/h, with a 1 km/h increase every 3 minutes until volitional exhaustion). In that study an initial moderate decrease in the oxygenation level in low-intensity exercise was observed followed by a rapid decrease in severe effort (greater for BB when compared to VL), suggesting that the O_2_ delivery to the less active muscle (BB) may be limited by the increase in ventilation in high-intensity running exercise. Although we investigated here the AO30 running effort unlike incremental treadmill running, it is possible that the acceleration of ventilatory responses in all-out exercise promoted a similar effect to that suggested by Rissanen^19^ on the TSI of the less active muscle.

Additionally, according to Secher and Volantis^64^ the greater TSI drop in the less active muscle as observed here in BB can be explained by the sympathetic flow induced by exercise, promoting vasoconstriction in this tissue and consequently a redirection of the blood flux to the more active muscle. A similar way was used by Shiroishi *et al*.^65^ to explain the decreased muscle oxygenation in the non-exercising limb during a graded leg cycling exercise, adopting NIRS and ultrasound measurements.

Kriel *et al*.^12,66^ suggested that Δ[HHb] is potentially unaffected by changes in perfusion, blood volume and arterial hemoglobin concentration in high-intensity exercise, in contrast to Δ[O_2_Hb]. So, due to these characteristics, we will focus our discussion on the delta of deoxyhemoglobin responses (Δ[HHb]). During AO30, we observed the drop in Δ[HHb] results in both BB and VL muscles. After this, Δ[HHb] presented stabilization to the vastus lateralis muscle oxygenation. At 28–30 seconds of exercise, the O_2_ utilization (signalized by Δ[HHb]) was higher in BB if compared to VL. We did not observe differences between Δ[tHb] in BB and VL during the A030 effort but a greater interindividual variation was shown in biceps brachii, which should be considered in future studies. Bhambhani^67^ based on the reports of Bae *et al*.^68^ suggested a significant contribution of the aerobic metabolism during high-intensity short duration exercise (such as the anaerobic Wingate test). Corroborating with these authors, our results showed an aerobic contribution during AO30 in tethered running, along with additional information about the significant participation of the less active muscle in this process.

Still comparing biceps brachii vs vastus lateralis oxygen responses, in a recent and interesting study, Willis *et al*.^13^ investigated the leg vs arm cycling repeated sprints with blood restriction and systemic hypoxia on peripheral and cerebral oxygenation. These authors observed greater changes in Δ[HHb] and Δ[tHb] in BB, suggesting that the arm is more responsible or sensitive to oxygen changes, especially induced by hypoxia, and it has a greater capacity to increase oxygen extraction in comparison to leg muscle. Although Willis *et al*.^13^ analysed the oxygenation of BB and VL, the purpose of those authors was not to investigate the responses of these muscles as more or less active in the same exercise, which makes comparison with our results difficult.

Another significant feature of our study was to monitor the metabolic and muscle oxygenation responses in a synchronized form up to 18 minutes after exercise by selecting investigation windows at every two minutes (Fig. 4). Thus, it was possible to observe which physiological responses return faster to the pre-exercise conditions, since recovery is an important component to improve physical training adaptations. Using these analyses, we observed that general responses (HR and blood lactate) did not return to baseline values after the recovery time chosen, at least in the active subjects studied here. However, the muscle oxygenation in the arm and leg are quickly adjusted in post-exercise, specially in BB, indicating that the use of NIRS technology showed it has higher sensitivity than classical exercise intensity markers, as well as can be used to improve the exercise monitoring and training prescription, as suggested by other research groups^30,39^.

As pertinently pointed out by Barstow^69^, the standardisation of protocols using NIRS is necessary. The attention to the instructions/limitations of each piece of equipment is important in order to minimise the possible intrinsic errors of the measurement; for example, the influence of the skin melanina and adipose tissue thickness^69^. In the present study, considerable differences were seen between BB and VL skinfold, which could account for some the of the differences seen in oxygenation responses. Aiming to minimise this methodological aspect, we use a NIRS devices that appears to be less sensitive to variations in adipose layer thickness^70^ applying the spatially resolved spectroscopy technique to analyse the TSI, and we choose the deeppest optode (40 mm) to investigated the [O_2_Hb], [HHb], and [tHb] in both muscles.

Focusing on muscle oxygenation responses after AO30, we observed differences between TSI and Δ[HHb] in the arm and leg muscles, especially in the initial minutes of exercise. BB showed lower TSI values immediately after AO30 than VL, but there was a quick and important reoxygenation in BB compared to VL (TSI in BB to from ~35% to ~70% within two minutes of recovery) (Fig. 4, Panel D). Our results are the opposite of Osawa *et al*.^14^, who investigated the recovery of BB after supramaximal cycling exercise with legs (140% of VO_2peak_ for 30 s and then no-load cycle exercise for 4 minutes). They observed that recovery of tissue oxygenation in biceps brachii did not occur immediately after effort and the reoxygenation in the arm was slower than in the leg. However, the exercise type adopted by Osawa *et al*.^14^ (cycling efforts for leg) and recovery process (arm in rest and leg in movement) are different that applied by us. In the light of our study, for high-intensity running exercise, the biceps brachii seems to play an important role during process recovery, which implies in future proposals of aerobic physical training to potentiate this muscle response.

In high-performance sports and physical training programmes, blood lactate concentration is widely used as a tool to quantify exercise intensity and to monitor training effects, given that this metabolite has the highest muscle production, release into the bloodstream^8^, and accumulates in response to exercise intensity^1^. Therefore, we proposed studying the relationship between blood lactate with muscle oxygenation parameters. Here, even though there was significant correlation between blood lactate concentration and the fatigue index in the time of the recovery, around the time to attain the peak of blood lactate, we only observed a significant correlation between peak [Lac] value to biceps brachii TSI and Δ[HHb] (in the 8^th^ and 10^th^ minute of the post-exercise period). In addition, when we applied Pearson’s product-moment test to analyse the correlations between blood lactate and peak, mean, and minimum muscle oxygenation responses, the most significant results were obtained with BB in exercise and recovery, but not with VL (the greatest blood lactate producer in our experimental design). These results reinforce an important contribution of the less active muscles on blood lactate responses, suggesting that more attention should be paid to this factor in exercise and training prescription. Corroborating with Willis *et al*.^13^, we also suggest that coaches and practitioners plan different training protocols for arms and legs to increase performance and physical adaptations, particularly for intensity running exercise.

Finally, in addition to other current measurements, such as HR, blood lactate, and VO_2_, we believe that very shortly peripherical oxygenation (including more or less active muscles) will be used to measure the internal load of training and recovery sessions, such as proposed here. We still agree that, despite some limitations^69^, wearable NIRS technology is a significant tool for monitoring the effects of training programs.

Due to this being a characterisation study about the kinetics of mechanical and physiological responses in all-out 30 seconds (AO30) tethered running followed by 18 minutes of passive recovery, some limitations must be considered. We did not use a gas analyser to investigate the oxygen uptake, even knowing the relevance of the VO_2_, as a continuous measurement in the experimental design adopted here. We considered that the use of the mask in this methodology could compromise our main goals. Another limitation of this study was sample composition, comprising only active male subjects. Future investigations with a similar protocol could be conducted with athletes of different modalities and also female participants.

In summary, here we provide the characterization of the mechanical, physiological and muscle oxygenation kinetics during and after high-intensity exercise, in particular improving the understanding of all-out tethered running. In addition to the important mechanical loading imposed, this kind of exercise promotes a high internal load during effort observed by physiological measurements, which do not return to rest values although after 18 minutes of passive recovery (at least by blood lactate and HR of the active subjects). On the other hand, muscle oxygenation responses presented faster adjustments (both during and after AO30) in a tissue-dependence manner, with very low TSI values observed in biceps brachii during the all-out effort. In addition, the significant correlations between peak and mean blood lactate with biceps brachii oxygenation indicate an integrative response of less active muscle oxygenation and metabolic events during and after high-intensity AO30 tethered running.
